# Association between gut microbiota and primary ovarian insufficiency: a bidirectional two-sample Mendelian randomization study

**DOI:** 10.3389/fendo.2023.1183219

**Published:** 2023-06-22

**Authors:** Jiahui Wang, Rong Luo, Xia Zhao, Di Xia, Yi Liu, Tao Shen, Yuanjiao Liang

**Affiliations:** ^1^ School of Medicine, Southeast University, Nanjing, China; ^2^ Department of Reproductive Medicine, Zhongda Hospital Affiliated to Southeast University, Nanjing, China

**Keywords:** primary ovarian insufficiency, gut microbiota, causal inference, Mendelian randomization study, genetics

## Abstract

**Background:**

Recent studies have indicated a potential correlation between intestinal bacteria and primary ovarian insufficiency (POI). However, the causal relationship between the gut microbiota (GM) and POI remains unclear.

**Methods:**

A bidirectional two-sample Mendelian randomization (MR) study was conducted to investigate the relationship between the GM and POI. Data on the GM were based on the MiBioGen consortium's summary statistics from the most comprehensive genome-wide association study meta-analysis to date (n=13,266), and POI data were obtained from the R8 release of the FinnGen consortium, containing a total of 424 cases and 181,796 controls. A variety of analytical methods, including inverse variance weighting, maximum likelihood, MR-Egger, weighted median, and constrained maximum likelihood and model averaging and Bayesian information criterion, were utilized to explore the connection between the GM and POI. The Cochran's Q statistics were used to evaluate the heterogeneity of instrumental variables. The MR-Egger and MR-pleiotropy residual sum and outlier (PRESSO) methods were used to identify the horizontal pleiotropy of instrumental variables. The MR Steiger test was used to evaluate the strength of causal relationships. A reverse MR study was performed to investigate the causal relationship between POI and the targeted GMs which were indicated to have a causal relationship with POI in the forward MR evaluation.

**Results:**

The inverse variance weighted analysis indicated that Eubacterium (hallii group) (odds ratio [OR]=0.49, 95% confidence interval [CI]: 0.26–0.9, P=0.022) and Eubacterium (ventriosum group) (OR=0.51, 95% CI: 0.27–0.97, P=0.04) had protective effects on POI, and Intestinibacter (OR=1.82, 95% CI: 1.04–3.2, P=0.037) and Terrisporobacter (OR=2.47, 95% CI: 1.14–5.36, P=0.022) had detrimental effects on POI. Results of the reverse MR analysis indicated that POI had no significant influence on the four GMs. No significant heterogeneity or horizontal pleiotropy was observed in the performance of the instrumental variables.

**Conclusion:**

This bidirectional two-sample MR study revealed a causal link between Eubacterium (hallii group), Eubacterium (ventriosum group), Intestinibacter, and Terrisporobacter and POI. Additional clinical trials are needed to gain a clearer understanding of the beneficial or detrimental effects of the GMs on POI and their mechanisms of action.

## 1 Introduction

Primary ovarian insufficiency (POI) is characterized by the absence of menstrual cycles caused by the disruption of ovarian activity before the age of 40 years. POI is distinguished by atypically high gonadotropin levels and low estrogen levels (1). It can lead to infertility in women, and most women with POI commonly display symptoms of menopause such as hot flashes, sleep disturbances, decreased sex drive, genital atrophy, painful intercourse, and emotional disruption. Furthermore, long-term complications resulting from a lack of estrogen, including osteoporosis, type 2 diabetes, and mental deterioration, may also occur in menopausal women (1). Although the causes of POI are unclear, hormonal imbalances, infections, environmental factors, medical procedures, and autoimmune conditions have been suggested as potential risk factors (2). POI is not a rare condition; according to statistics, 0.01% of women aged <20 years, 0.1% of women aged <30 years, and 1% of women aged <40 years have POI (3). Although spontaneous ovulation occurs frequently, only 5% of patients may spontaneously become pregnant, and the majority of POI cases result in lifelong loss of fertility (4). Thus, POI has received worldwide attention, especially among young women, because of its negative effects and the strong desire of patients to conceive. Studies have reported that stabilization of the gut microbiota (GM) composition significantly influences women’s reproductive health (5), and may lead to endometriosis (6), polycystic ovary syndrome (7), and POI, owing to its ability to regulate sex hormones, produce inflammatory agents, affect immunity, and stabilize metabolism. These studies provide a new perspective on the pathogenesis of POI, which may have great research potential and applied research value in providing new prevention, therapy, and management tools for POI.

Recently, owing to advancements in gene sequencing technology, microflora identification has been expedited and is being extensively utilized in the testing domain. The link between the GM and POI has recently attracted the attention of researchers in related fields. Several studies have suggested that the composition and structure of the GMs in patients with POI differ from those in healthy patients. Wu et al. have reported that the phyla *Bacteroidetes*, *Butyricimonas*, *Dorea*, *Lachnobacterium*, and *Sutterella* were considerably enriched in patients with POI; however, the phyla *Firmicutes*, *Bulleidia*, and *Faecalibacterium* were more prevalent among healthy women. These changes in the GMs in patients with POI were also closely correlated with the levels of follicle-stimulating hormone (FSH), luteinizing hormone (LH), estradiol (E2), anti-Müllerian hormone, and the FSH/LH ratio (8). In a mouse model of POI induced by 4-vinylcyclohexene diepoxide, Cao et al. observed a significant increase in the alpha diversity of the intestinal flora in POI mice compared to that in normal mice, with lower abundances of *Helicobacter*, *Odoribacter*, and *Alistipes* and higher abundances of *Clostridium* XIVa, *Barnesiella*, *Bacteroides*, and *Mucispirillum* (9). Lin et al. have reported that GMs were significantly altered in mice with cyclophosphamide (CTX)-induced POI, with a decrease in the abundance of *Akkermansia* and a significant increase in *Lactobacillus*, and that Fisetin attenuated CTX-induced ovarian damage by modulating the GMs in a manner that decreased CCR9+/CXCR3+/CD4+ T-lymphocyte counts and IL-12 secretion (10). However, current research on POI and GMs is relatively limited. Recent research includes case–control studies, which are limited in their ability to establish causality. Furthermore, effectively controlling for confounding factors such as age, environment, dietary habits, and lifestyle can be difficult in an observational study (11), which may influence the relationship between GMs and POI. These variables limit the causal link between the GMs and the POI.

Therefore, we used a bidirectional Mendelian randomization (MR) approach to explore a possible causal relationship between GM and POI. Magnetic resonance imaging is a powerful genetic epidemiological tool based on Mendel’s independent assignment theorem. MR uses genetic variation and single nucleotide polymorphism s(SNPs) as instrumental variables (IV) to assess the causality of risk factors for complex diseases. According to Mendel’s laws of inheritance, alleles are passed randomly from parent to offspring at conception, similar to the principle of random assignment in randomized controlled trials. Thus, MR can avoid the pitfalls of observational studies, such as bias and reverse causality, and can infer causal relationships between complex diseases (12). To date, MR has been extensively used to explore the association between GMs and various medical conditions, such as metabolic (13), autoimmune (14), mental (15), and pregnancy complications (16). In this study, a bidirectional MR analysis was performed to assess the causal relationship between GMs and POI using combined data on genetic variations from the MiBioGen and FinnGen consortia via genome-wide association studies. The application of a bidirectional MR design made our results more robust to confounding factors and reverse causality.

## 2 Methods

### 2.1 Data source

SNPs of GMs selected as IVs were derived from a genome-wide association study (GWAS) meta-analysis of the MiBioGen consortium (17, 18) with 18,340 individuals, including 122,110 associated SNPs. This was a large-scale multi-ethnic GWAS that recruited 24 population-based cohorts and identified 211 GMs; the majority of individuals had European origins. These cohorts included participants from the USA, Canada, Israel, South Korea, Germany, Denmark, the Netherlands, Belgium, Sweden, Finland, and the UK. Among these cohorts, 20 comprised samples from individuals of a single ancestry, including European (16 cohorts, N=13,266), Middle Eastern (one cohort, N=481), East Asian (one cohort, N=811), American Hispanic/Latin (one cohort, N=1,097), and African American (one cohort, N=114) populations. Additionally, four cohorts had multiple ancestries, comprising 2,571 participants. Further details of the 24 cohorts have been reported in a previous publication (17). The lowest taxonomic level examined in this study was the genus; 131 genera with an average abundance of >1% were identified, of which 12 were unidentified (17). Consequently, 119 genus-level GMs were selected for analysis in this study. Additionally, GWAS summary statistics related to POI risk were extracted from the FinnGen Consortium R8 release data, which included 424 cases and 181,796 controls of Finnish adult female participants (19, 20). The FinnGen study is a research project that integrates genetic data from Finnish biobanks with health records sourced from Finnish health registries. This study used International Classification of Diseases codes to establish the research endpoints. During the course of the study, sex, age, genotyping batch, and the first ten main components were corrected (19). As the present study utilized publicly available summary data, no additional ethical approval or participant consent was required.

### 2.2 Instrumental variables

To guarantee the authenticity and accuracy of the findings concerning the connection between the GM and POI risk, the following quality control procedures were applied to select optimal instrument variables: (1) the association between SNP and POI reached the locus-wide significance level (1 × 10^−5^), and SNPs with a *P*-value less than this level were selected as IVs; (2) we excluded SNPs with linkage disequilibrium (*R* ^2^ < 0.1 and clumping window size = 500 kb), ensuring that the IVs are independent of each other, to reduce the effects of linkage disequilibrium that violates random allele assignment, which is in line with previous studies (21); (3) when palindromic SNPs were present, allele frequency information was used to infer the forward-strand alleles; and (4) we calculated the F-statistic for each SNP, excluding weak IVs (F ≤ (F), to guarantee the robustness of the association between the IVs and exposure factors (F = β^2^exposure/SE^2^exposure).

### 2.3 Statistical analysis

We employed a bidirectional two-sample MR approach to separate GMs and POI data from the GWAS. We applied various high-efficiency methods, including inverse variance weighted (IVW), constrained maximum likelihood and model averaging and Bayesian information criterion (cML-MA-BIC), maximum likelihood (ML), MR-Egger regression, and weighted median, to identify a a correlation between GMs and POI. The IVW technique, the most widely employed MR technique, was the principal method of analysis used in this study. The IVW method is a causal inference method based on genetic summary data. The Wald ratios corresponding to individual SNPs were calculated by dividing the effect value between the SNP and POI by the effect value between the SNP and GMs. Wald ratios for all SNPs were weighted together to assess the genetic association between GMs and the risk of developing POI. The IVW method can obtain accurate results when all IVs in the analysis are assumed to have solid validity. However, the results exhibited errors in the presence of invalid IVs. This is because the IVW method requires all SNPs to be valid IVs; if one of the IVs is invalid, the IVW method may produce inaccurate estimates. Therefore, when using the IVW method, verifying the validity of all indicator variables is necessary to avoid errors. Although the IVW method requires all SNPs to be valid IVs, the weighted median method can generate an accurate assessment of the causal effect, even when up to 50% of the data being analyzed are derived from invalid IVs (22). The ML method, also known as the most approximate estimation method, is a theoretical point-estimation method. Compared to the IVW method, the ML method has the advantage of a lower standard error, and its results are unbiased if there is no heterogeneity or horizontal polymorphism (23). The MR-Egger regression method is a multi-IV MR method based on pooled data modified from IVW. In contrast to the IVW method, the MR-Egger regression method only needs to satisfy the IVs pleiotropic effect independent of the association between IVs and exposure factors (instrument strength independent of direct effect, InSIDE) assumption and the no-measurement error assumption, which are less demanding than the three core assumptions of IVs. Simultaneously, this technique can identify and counteract pleiotropy bias. Therefore, utilizing MR-Egger regression can maintain the accuracy of MR techniques in studies with numerous genetic variants as IVs (22). The cML-MA-BIC method, an MR technique that utilizes ML and model averaging without depending on the InSIDE assumption, was employed to manage both correlated and uncorrelated pleiotropic effects (24). This study was primarily based on the IVW approach, with four other techniques enhancing the results.

To guarantee the accuracy of the MR outcomes, we conducted diversity and sensitivity assessments. Cochran’s Q test was used to determine the heterogeneity among SNPs (23), and if the Q-P was >0.05, no significant heterogeneity was observed among the IVs. We then applied the MR-Egger method (25) to identify horizontal pleiotropy among the IVs, and *P*>0.05 indicates no horizontal pleiotropy. Additionally, we evaluated horizontal pleiotropy and outliers using the MR-pleiotropy residual sum and outlier (MR-PRESSO) global test (26). Furthermore, we used the “leave-one-out” method to demonstrate visually whether a single SNP drove the main causal relationship. Possible pleiotropic effects of the SNPs were searched using the Phenoscanner website (http://www.phenoscanner.medschl.cam.ac.uk/) (27). Additionally, the MR Steiger directionality test was used to further evaluate the correlation between exposure and outcome (28). To determine the correlation between GMs and POI, we performed a reverse MR analysis to GMs previously identified by forward MR analysis as having a causal link to POI. The settings and methodologies were in accordance with those for forward MR.

A strong causal relationship between the GMs and POI was considered when the following criteria were met: 1) the IVW method demonstrated a significant difference (*P* < 0.05); 2) the five methods provided consistent estimations; 3) the Cochran’s Q test, MR-Egger test, and MR-PRESSO global test had no significance (*P* > 0.05) (29); and 4) the MR–Steiger directionality tests indicated TRUE. Statistical analyses were performed using R version 4.2.2.

## 3 Results

### 3.1 Selection of IVs

In total, 1,269 SNPs were selected as IVs for 119 bacterial genera in accordance with the specified selection criteria. The F-statistics of the SNPs included in the analysis were >10, demonstrating that the IVs used were robust. Thus, no weak bias was observed in the results, and the findings of this study were acceptable (**Supplementary Table S1**).

### 3.2 Two-sample MR analysis

We identified four GM genera (*Eubacterium hallii*, *Eubacterium ventriosum*, *Intestinibacter*, and *Terrisporobacter*) related to POI after setting a standard in which the IVW method demonstrated a significant difference (*P* < 0.05), and the five methods indicated consistent directions.

**Figure 1:**
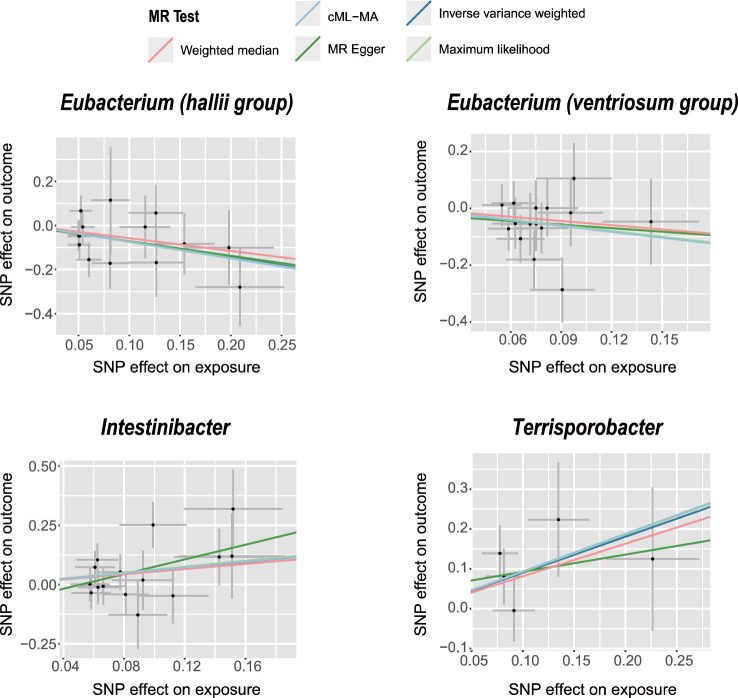
Scatter plots of significant causality of the GM and POI. Scatter plot of the effect size and 95% confidence interval of each SNP on GM and POI risk. The horizontal axis reflects genetic effect of each SNP on GM. The vertical axis represents the genetic effect of each SNP on POI risk.

**Figure 2:**
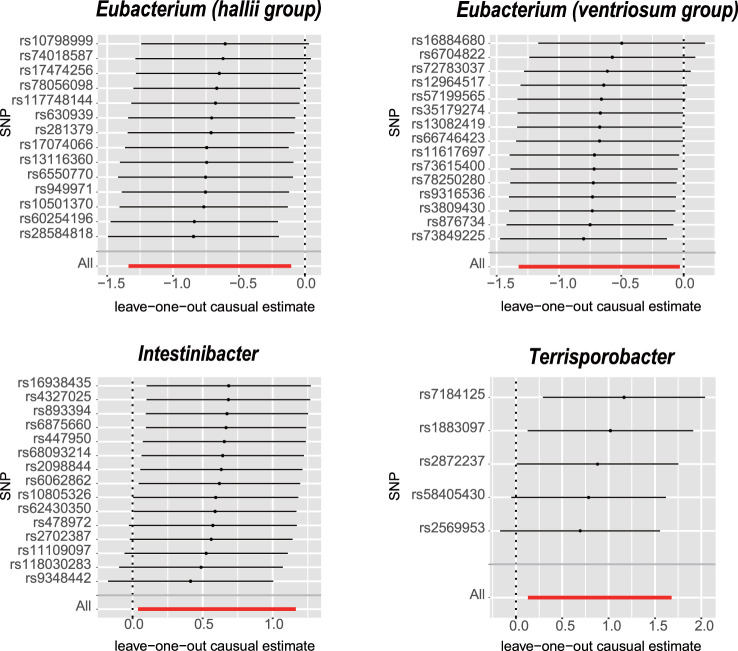
Leave-one-out analysis for the impact of individual SNPs on the association between GM and POI risk. By leaving out exactly one SNP, it demonstrates how each individual SNP influences the overall estimate.

The IVW analysis revealed that *E. hallii* (odds ratio [OR]=0.49, 95% confidence interval [CI]: 0.26–0.9, *P*=0.022) and *E. ventriosum* (OR = 0.51, 95% CI:0.27–0.97, *P*=0.04) had a negative relationship with the risk of POI. The IVW analysis showed that *Intestinibacter* (OR=1.82, 95% CI:1.04–3.2, *P*=0.037) and *Terrisporobacter* (OR=2.47, 95% CI:1.14–5.36, *P*=0.022) were positively associated with the risk of POI (**Table 1**). Cochran’s Q test demonstrated no heterogeneity between the IVs. The results of the MR-Egger regression intercept and MR-PRESSO tests revealed little evidence of horizontal pleiotropy in the IVs. The scatter plots (**Figure 1**) and leave-one-out plots (**Figure 2**) did not demonstrate any potential outliers in the IVs. No SNPs were identified as outliers in the MR-PRESSO analysis. Additionally, all the MR Steiger directionality tests indicated a robust direction from the GMs to the POI for all outcomes (**Table 2**). The MR statistics for the 119 GMs on POI are presented in detail in **Supplementary Tables S2–7**.

**Table 1:** Summary results of MR (Target GM on POI).

					Cochran Q-test		Directional pleiotropy		
Exposure	Method	NSNPs	OR(95% CI)	*P*	*P*	I^2^ (%)	Egger intercept *(P*)	MRPRESSO global test RSSobs (*P*)	Correct Causal direction
*Eubacterium (hallii group)*	IVW	14	0.49(0.26-0.9)	2.20E-02	0.77	0.00	-5.72E-03(0.92)	10.332(0.80)	TRUE
*Eubacterium (hallii group)*	cML-MA-BIC	14	0.47(0.25-0.89)	2.10E-02					
*Eubacterium (hallii group)*	Maximum likelihood	14	0.48(0.25-0.9)	2.20E-02					
*Eubacterium (hallii group)*	MR Egger	14	0.52(0.14-1.86)	0.33					
*Eubacterium (hallii group)*	Weighted median	14	0.56(0.24-1.33)	0.19					
*Eubacterium (ventriosum group)*	IVW	15	0.51(0.27-0.97)	4.00E-02	0.76	0.00	-0.02(0.86)	11.482(0.78)	TRUE
*Eubacterium (ventriosum group)*	cML-MA-BIC	15	0.5(0.26-0.98)	4.40E-02					
*Eubacterium (ventriosum group)*	Maximum likelihood	15	0.51(0.26-0.99)	4.70E-02					
*Eubacterium (ventriosum group)*	MR Egger	15	0.66(0.04-11.77)	0.78					
*Eubacterium (ventriosum group)*	Weighted median	15	0.61(0.26-1.44)	0.26					
*Intestinibacter*	IVW	15	1.82(1.04-3.2)	3.70E-02	0.53	0.00	-0.08(0.31)	14.897(0.55)	TRUE
*Intestinibacter*	cML-MA-BIC	15	1.83(1.02-3.3)	4.40E-02					
*Intestinibacter*	Maximum likelihood	15	1.87(1.05-3.33)	3.30E-02					
*Intestinibacter*	MR Egger	15	4.71(0.75-29.57)	0.12					
*Intestinibacter*	Weighted median	15	1.72(0.75-3.94)	0.2					
*Terrisporobacter*	IVW	5	2.47(1.14-5.36)	2.20E-02	0.57	0.00	0.05(0.70)	4.554(0.62)	TRUE
*Terrisporobacter*	cML-MA-BIC	5	2.56(1.13-5.82)	2.40E-02					
*Terrisporobacter*	Maximum likelihood	5	2.54(1.13-5.72)	2.40E-02					
*Terrisporobacter*	MR Egger	5	1.54(0.15-15.89)	0.74					
*Terrisporobacter*	Weighted median	5	2.26(0.81-6.31)	0.12					

MR, Mendelian randomization; GM, gut microbiota; POI, primary ovarian insufficiency;IVW, inverse variance weighted; NSNPs, number of single nucleotide polymorphisms; OR, odds ratio; CI, confidence interval; RSSobs, residual sum of squares of observations.

**Table 2:** Summary results of MR (POI on target GM).

					Cochran Q-test		Directional pleiotropy		
Outcome	Method	NSNPs	OR(95% CI)	*P*	*P*	I^2^(%)	Egger intercept *(P*)	MRPRESSO global test RSSobs (*P*)	Correct Causal direction
*Eubacterium (hallii group)*	IVW	5	1.03 (1.00-1.06)	0.05	0.61	0.00	0.023(0.44)	4.397(0.65)	TRUE
*Eubacterium (hallii group)*	cML-MA-BIC	5	1.03 (1.00-1.06)	0.05					
*Eubacterium (hallii group)*	Maximum likelihood	5	1.03 (1.00-1.06)	0.05					
*Eubacterium (hallii group)*	MR Egger	5	0.98 (0.87-1.10)	0.74					
*Eubacterium (hallii group)*	Weighted median	5	1.03 (0.99-1.06)	0.17					
*Eubacterium (ventriosum group)*	IVW	5	0.99 (0.96-1.03)	0.74	0.27	22.74	-0.001(0.98)	8.163(0.35)	TRUE
*Eubacterium (ventriosum group)*	cML-MA-BIC	5	0.99 (0.96-1.02)	0.69					
*Eubacterium (ventriosum group)*	Maximum likelihood	5	0.99 (0.96-1.02)	0.7					
*Eubacterium (ventriosum group)*	MR Egger	5	1.00 (0.85-1.17)	0.97					
*Eubacterium (ventriosum group)*	Weighted median	5	0.99 (0.96-1.03)	0.75					
*Intestinibacter*	IVW	5	1.00 (0.96-1.04)	1	0.28	21.10	0.033(0.42)	7.302(0.37)	TRUE
*Intestinibacter*	cML-MA-BIC	5	1.00 (0.97-1.03)	0.98					
*Intestinibacter*	Maximum likelihood	5	1.00 (0.97-1.03)	1					
*Intestinibacter*	MR Egger	5	0.93 (0.80-1.08)	0.43					
*Intestinibacter*	Weighted median	5	0.99 (0.95-1.04)	0.73					
*Terrisporobacter*	IVW	5	1.01 (0.94-1.08)	0.8	0.05	57.97	0.106(0.09)	15.947(0.09)	TRUE
*Terrisporobacter*	cML-MA-BIC	5	1.01 (0.96-1.06)	0.76					
*Terrisporobacter*	Maximum likelihood	5	1.01 (0.96-1.06)	0.68					
*Terrisporobacter*	MR Egger	5	0.80 (0.67-0.97)	0.1					
*Terrisporobacter*	Weighted median	5	0.99 (0.93-1.05)	0.66					

MR, Mendelian randomization; POI, primary ovarian insufficiency; GM, gut microbiota; IVW, inverse variance weighted; NSNPs, number of single nucleotide polymorphisms; OR, odds ratio; CI, confidence interval; RSSobs, residual sum of squares of observations.

The outcomes of the reverse MR study indicated no conclusive evidence of a causal relationship between POI and the four GMs (**Table 2** and Additional File 1; **Table S9**). Cochran’s Q test, MR-Egger, and MR-PRESSO analyses (**Table 2** and Additional file 1: **Tables S10–12**) did not indicate substantial heterogeneity or horizontal pleiotropy. The results of the MR Steiger directionality tests for the POI on the four GMs were TRUE (**Table 2** and Additional file 1: **Table S13**).

## 4 Discussion

We employed a bidirectional two-sample MR analysis to examine the causal relationship between GMs and POI. To the best of our knowledge, this is the first large-scale MR analysis to investigate the correlation between GMs and POI at the gene level using the largest and most recent GWAS data, which may provide useful advice for the prevention, treatment, and management of POI by concentrating on certain GMs. The results of this study indicate that *E. hallii* and *E. ventriosum* provide protection, whereas *Testinibacter* and *Terrisporobacter* have detrimental effects on POI.

The GMs are symbiotic microorganisms that colonize the human gut and are known as the “second genome” because of their abundance, diversity, and genetic information. These GMs form an interdependent symbiotic relationship with the host, affecting normal physiological functions and disease susceptibility (30). The GM plays an important role in human growth and development, metabolism, immunity, and other pathophysiological processes, including promoting the maturation of host immune system. It inhibits the overgrowth of pathogens, influences host cell proliferation and blood vessel formation, regulates intestinal endocrine function, neural signaling, energy sources, vitamin, and neurotransmitter synthesis (29, 31–33). The intestinal flora not only has various effects on the intestinal environment, but also regulates distal tissues and organs. Many scholars have proposed the concepts of “brain–GM axis” (34), “gut–liver axis” (35), “gut–kidney axis” (36), “estrogen–GM axis” (37), and “gut–brain–ovary axis” (38).

POI is an intricate multifactorial disorder associated with autoimmune conditions, infections, enzyme deficiencies, hereditary changes, environmental influences, and iatrogenic intervention. Several observational studies have reported a relationship between the GMs and POI. Recent studies have suggested that the connection between GMs and POI is mostly related to autoimmunity and its role in the control of sex hormones, with some studies indicating a potential link with metabolic diseases. However, the GMs may have a direct or indirect role in the control of sex hormones (39), as well as inflammation, production of immune-related cytokines, including Treg, IFN-γ, and Th17, and regulation of the body’s metabolic processes to influence POI (40).

*Eubacterium* is a type of bacteria that is highly diverse in terms of phylogeny and forms an integral part of human GMs (41). Several *Eubacterium* genus produce butyrate and propionate, which are short-chain fatty acids (SCFAs) that play pivotal roles in maintaining energy balance, stimulating colonic activity, modulating the immune system, and reducing inflammation in the intestine. *Eubacterium* genus are capable of transforming bile acids and cholesterol in the gut, which, in turn, helps maintain the gut ecosystem’s equilibrium. The altered balance of GMs and changes in *Eubacterium* genus have been associated with numerous health problems in humans (41). *E. hallii* is a member of the *Lachnospiraceae* family of the phylum *Firmicutes* and produces butyrate, an SCFA that has beneficial effects on metabolism and inflammation. *E. hallii* has been reported to improve endocrine diseases, diabetes, dyslipidemia, and other conditions linked to insulin resistance (42). *E. ventriosum* belongs to the *Lachnospiraceae* family of the phylum *Firmicutes* and produces SCFAs. Liu et al. have suggested that *E. ventriosum* was associated with a lower risk of inflammatory bowel disease and ulcerative colitis. Although no previous studies have established a connection between *E. hallii*, *E. ventriosum*, and POI, Wu et al. have reported that, the phylum *Firmicutes* was more prevalent among healthy women than among patients with POI (8), which is consistent with our findings; however, deep investigations are needed to confirm this hypothesis. *Intestinibacter* belongs to the family *Clostridiaceae* and is a Gram-positive, rod-shaped, non-motile, spore-forming bacterium that lives in the gut (43). Previous studies on *Intestinibacter* are limited. *Terrisporobacter* is a Gram-positive, spore-forming, anaerobic bacterium belonging to the family *Peptostreptococcaceae*. *Terrisporobacter* can cause infections in humans, especially in postoperative patients with comorbidities, such as cirrhosis, brain abscesses, bone and joint infections, and bloodstream infections (44).

This study has several strengths. Most previous studies exploring the relationship between GMs and POI utilized animal models (9, 10). Research conducted on patients with POI by collecting fecal samples yielded only cross-sectional data, thereby precluding the possibility of establishing a cause–effect relationship between GM and POI (8). This study utilized MR to analyze the connection between the GM and POI in humans, thereby minimizing the impact of extraneous variables and ensuring a valid cause–effect relationship. We used GM summary statistics from the MiBioGen consortium’s most extensive GWAS meta-analysis and POI summary statistics from FinnGen’s R8 release data to ensure the reliability of our instruments and the latest data for the MR study. The MR-PRESSO and MR-Egger tests were used to detect and exclude horizontal pleiotropy. In addition, the cML-MA-BIC method was employed to eliminate bias from correlated and uncorrelated pleiotropies. To avoid bias, this MR study employed summary-level data that did not overlap with exposure and results.

However, this study has some limitations that should be considered when interpreting the findings. First, analyzing nonlinear correlations was difficult because the analysis was based on summarized figures rather than raw data. Second, the GWAS for GM did not exclusively focus on female participants. Although the analysis considered dissimilarities between sexes, omitting any genetic variants located on the sex chromosomes, circumventing any potential prejudice caused by sex was not feasible (24). Third, we restricted our inquiry to GWASs in Europe; therefore, whether the findings of this study can be extended to individuals of non-European ancestry owing to genetic disparities between races is uncertain (45). Finally, further exploration of the correlation between the GM and POI at the species level was not possible because exposure data were available only at the genus level.

## 5 Conclusion

*E. hallii* and *E. ventriosum* have protective effects against POI, whereas *Intestinibacter* and *Terrisporobacter* have detrimental effects on POI. Additional studies should be conducted to elucidate the potential protective or detrimental effects of GM on POI and the mechanisms involved. Despite the lack of evidence indicating that POI has an impact on GM, it is conceivable that it might have an effect, thereby necessitating further investigation to ascertain this fact.

## Data availability statement

The original contributions presented in the study are included in the article/**Supplementary Material**. Further inquiries can be directed to the corresponding author.

## Ethics statement

Ethical approval was not provided for this study on human participants because this research has been conducted using published studies and consortia providing publicly available summary statistics. All original studies have been approved by the corresponding ethical review board. In addition, no individual-level data was used in this study. Therefore, no new ethical review board approval was required. Written informed consent to participate in this study was provided by the participants’ legal guardian/next of kin.

## Author contributions

Conception and design: JW, RL, XZ, DX, and YJL. Data collection and assembly: JW, YJL, and TS. Data analysis and interpretation: JW, RL, XZ, DX, and TS. Manuscript writing: JW. Final approval of the manuscript: All authors.
